# Thermodynamic and Transport Properties of Tetrabutylphosphonium Hydroxide and Tetrabutylphosphonium Chloride–Water Mixtures via Molecular Dynamics Simulation †

**DOI:** 10.3390/polym12010249

**Published:** 2020-01-20

**Authors:** Brad Crawford, Ahmed E. Ismail

**Affiliations:** Department of Chemical and Biomedical Engineering, West Virginia University, Morgantown, WV 26505, USA

**Keywords:** water, biofuel, biomass, molecular dynamics simulation, ionic liquid, cellulose, diffusion

## Abstract

Thermodynamic, structural, and transport properties of tetrabutylphosphonium hydroxide (TBPH) and tetrabutylphosphonium chloride (TBPCl)–water mixtures have been investigated using all-atom molecular dynamics simulations in response to recent experimental work showing the TBPH–water mixtures capability as a cellulose solvent. Multiple transitional states exist for the water—ionic liquid (IL) mixture between 70 and 100 mol% water, which corresponds to a significant increase in water hydrogen bonds. The key transitional region, from 85 to 92.5 mol% water, which coincides with the mixture’s maximum cellulose solubility, reveals small and distinct water veins with cage structures formed by the TBP^+^ ions, while the hydroxide and chloride ions have moved away from the P atom of TBP^+^ and are strongly hydrogen bonded to the water. The maximum cellulose solubility of the TBPH–water solution at approximately 91.1 mol% water, appears correlated with the destruction of the TBP’s interlocking structure in the simulations, allowing the formation of water veins and channeling structures throughout the system, as well as changing from a subdiffusive to a near-normal diffusive regime, increasing the probability of the IL’s interaction with the cellulose polymer. A comparison is made between the solution properties of TBPH and TBPCl with those of alkylimidazolium-based ILs, for which water appears to act as anti-solvent rather than a co-solvent.

## 1. Introduction

The cellulose polymer contained in plant biomass, particularly waste biomass, is one of the most abundant materials on earth, yet it remains largely untapped as an industrial feedstock [1]. If cellulose in wastes such as cornstalks post-growing season, dead trees, and sawmill scraps can be economically extracted from the biomass, the resulting biomass feedstock could be used for both commodity and specialty chemical production. In this regard, ionic liquids (ILs) have shown promise as solvents for the dissolution of cellulosic biomass [1,2,3,4,5,6,7,8,9]. ILs are a class of materials that possess low melting points, are non-flammable and nearly non-volatile, and demonstrate chemical and thermal stability under a range of operating conditions, making them well suited for industrial processes [1,2,3,4,5,6,7,8,9]. For example, alkylimidazolium-based ILs such as 1-butyl-3-methylimidazolium chloride ([BMIM]Cl) have been studied and shown to be effective; however, elevated temperatures (323 to 373 K) are required to dissolve high concentrations [1,5,6,7]. The need for elevated temperatures increases the energy input, adding costs and potentially negating any benefits of the process. At moderate temperatures, alkylimidazolium-based ILs appear to be extremely sensitive to the presence of water [5,6], where, for example, 1-ethyl-3-methylimidazolium chloride ([EMIM][Cl]) demonstrates highly unusual, concentration-dependent artifacts in X-ray diffraction analyses of pre-treated biomass as a function of water concentration [8]. Similarly, for the temperatures 343 K and 373 K, pure [BMIM]Cl can dissolve 3.0 wt% and 10 wt% of cellulose, respectively; however, at 363 K and 368 K, a 20 wt% (70.8 mol%) aqueous mixture of [BMIM]Cl can only dissolve a maximum of 1.1 wt% and 0 wt% cellulose respectively [1,5,6]. Given that biomass can naturally contain as much as 25 wt% water, it is essential that any chemical species chosen for biomass dissolution be able to tolerate the presence of water if it is to be a viable industrial solvent for cellulose. While pre-processing such as drying of the biomass can be performed, these add costs to industrial processes. Even natural drying methods that do not require large energy inputs, such as using ambient air or sunlight, still require either additional pre-processing or large storage areas, thereby increasing the capital cost and decreasing its commercial viability. Because many alkylimidazolium ILs show optimal dissolution at elevated temperatures and low water content, their viability as industrial solvents is reduced [9,10].

**Figure 1:**
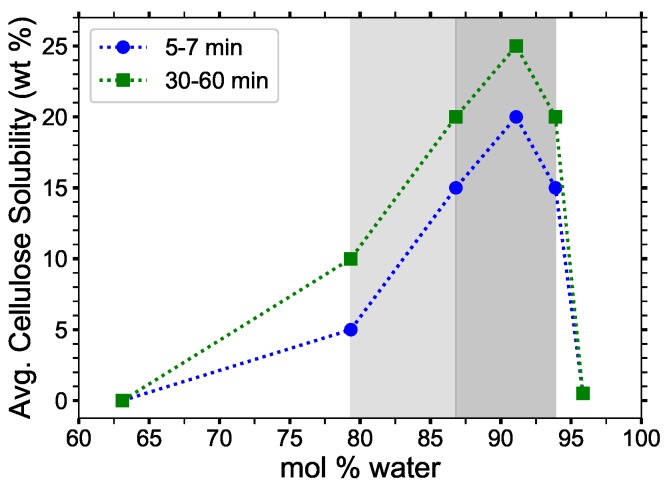
Cellulose solubility in tetrabutylphosphonium hydroxide (TBPH)-water solution as a function of water concentration for *p* = 1 atm and *T* = 298.15 K The experimental cellulose solubility ranges are shown throughout the paper, and were broken down into two different ranges. Light gray shading shows where cellulose is soluble, but not ideal (79.4 to 86.8 mol% water). Dark gray shading shows the maximum cellulose solubility range (86.8 to 93.9 mol% water). Data from Abe et al. [2].

**Figure 2:**
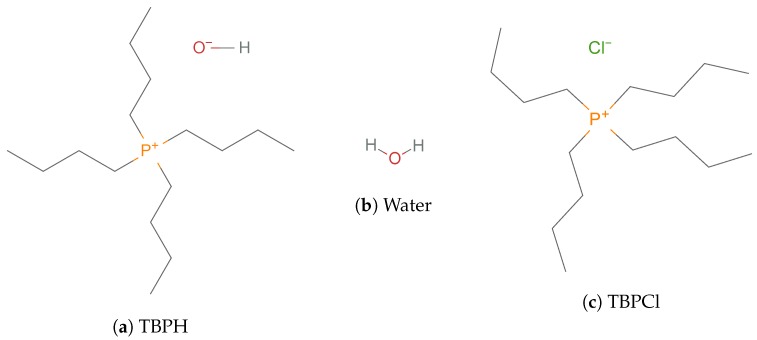
Chemical structures of the molecules [34].

Recently, tetrabutylphosphonium hydroxide (TBPH) has demonstrated a strong ability to dissolve cellulose with high water concentrations [2]. TBPH–water has also been shown to operate across this wide range of concentrations while retaining its ability to dissolve cellulose of a relatively constant amount [2]. Specifically, aqueous solutions of the IL TBPH with between 86.8 and 93.9 mol% water (30 to 50 wt% water) can dissolve at least 20 wt% cellulose at room temperature; this dissolution process has also been observed to be only moderately sensitive to time, where at least 15 wt% cellulose can be dissolved in under 7 min, further making this an attractive solvent for industrial applications [2]. As compared to alkylimidazolium-based ILs, the symmetrically branched structure of the TBP molecule seemingly contributes to the increased dissolution power of the TBPH–water system (see Figure 1 and Figure 2), however, there is little work exploring the mechanism that underlies TBPH’s exceptional capability to dissolve the cellulose polymer under hydrated conditions.

Here, the chemical properties of TPBH–water and tetrabutylphosphonium chloride (TBPCl)–water solutions are investigated at varying water concentrations to identify the source of their unique, effective cellulose dissolving characteristics. Elucidating the properties that allow cellulose dissolution at low temperature and in the presence of high water concentrations is an essential step to complete the larger goal, being the design and development of more economical solvents for cellulose dissolution and fuel production. Molecular dynamics (MD) simulations have been performed herein to determine the structural, physical, and transport properties of aqueous mixtures of the TBPH–water and TBPCl–water solutions throughout the range of cellulose solubility. Physical properties examined include density, excess molar volume, hydrogen bonding, and radial distribution functions (RDFs). Thermodynamic properties include the enthalpy of mixing, heat capacity (*c_p_*), and thermal expansivity (*α_p_*), and transport properties include the generalized diffusion coefficient and the anomalous diffusion exponent. The paper is organized as follows. First, the methodology and force field validation are presented. Next, the structural, thermodynamic, and diffusive properties of the simulations are presented and correlated with experimental solubility, comparing the behaviors of TBPH, TBPCl, and imidazolium-based ILs. Finally, a discussion of the results is presented along with the concluding remarks.

## 2. Methodology

Here, classical (i.e., non-reactive) molecular dynamics simulations are performed using the large-scale atomic/molecular massively parallel simulator (LAMMPS) program (version 22-Feb-2018) [11]. Initial configurations were generated using the PACKMOL software and visualizations were completed using visual molecular dynamics (VMD) [12,13]. The force field for the tetrabutylphosphonium (TBP^+^) cation is taken from Zhou et al. [14], the chloride anion is taken from Sambasivarao et al. [15], and the force field for the hydroxide anion from Våcha et al. [16]. The four-site transferrable intermolecular potential (TIP4P)/2005 model was used for water [17]. The potential energy *E*^total^ equation is based on the assisted model building with energy refinement (AMBER) potential [18]:

(1) $$\begin{array}{c} E_{\mathrm{total}}=\sum_{\mathrm{bonds}}K_{r}{\left(r-r_{0}\right)}^{2}+\sum_{\mathrm{angles}}K_{\theta }{\left(\theta -\theta_{0}\right)}^{2}+\sum_{\mathrm{torsions}}\frac{K_{\phi }}{2}\left(1+\cos\left(n\phi -\gamma \right)\right)\\ +\sum_{i=1}^{N}\sum_{j=i+1}^{N}\left\{4\epsilon_{ij}\left[{\left(\frac{\sigma_{ij}}{r_{ij}}\right)}^{12}-{\left(\frac{\sigma_{ij}}{r_{ij}}\right)}^{6}\right]+\frac{q_{i}q_{j}}{r_{ij}}\right\}, \end{array}$$

where $K_{r}$, $K_{\theta }$, and $K_{\phi }$ are the bond, angle, and torsion constants; *r*, θ, and ϕ are the measured bond lengths, bond angles, and torsion angles; $r_{0}$, $\theta_{0}$, and γ are the equilibrium bond lengths, bond angles, and torsion angles; $\epsilon_{ij}$ and $\sigma_{ij}$ are the Lennard-Jones potential parameters; $r_{ij}$ is the measured separation between atoms *i* and *j*; and $q_{i}$ and $q_{j}$ are the charges of atoms *i* and *j*. The Lorentz-Berthelot mixing rules were used for the mixed $\epsilon_{ij}$ and $\sigma_{ij}$ parameters, respectively [19,20]:

(2) $$\begin{array}{c} \epsilon_{ij}=\sqrt{\epsilon_{ii}\epsilon_{jj}},\\ \sigma_{ij}=\frac{\left(\sigma_{ii}+\sigma_{jj}\right)}{2} \end{array}$$

Both isobaric-isothermal (*NPT*) and constant volume-isothermal (*NVT*) production runs were conducted using approximately 10,000 atoms (see Table 1). All simulations used the velocity Verlet algorithm [21]. A dual split reversible reference system propagator algorithms (RESPA) method was used with an inner cutoff of 4 Å and outer cutoff of 5 Å [22]. The inner cutoff had a timestep of 2 fs and covered the bond, angle, dihedral, improper, and pair-inner terms. The outer cutoff had a timestep of 4 fs and covered the pair-outer and Coulombic terms. The short-range electrostatic and dispersion force cutoffs of 10 Å were selected for all non-bonded atoms. The particle–particle–particle–mesh (PPPM) method was used for the long-range electrostatics, using an accuracy of 10^−5^ [23]. Long range dispersion forces were calculated using the Isele-Holder method for the Lorentz-Berthelot or no mixing rules, using a real space accuracy of 10^−4^ and a kspace accuracy of 2 × 10^−3^ [19,20,24]. All simulations used the Nosé-Hoover system to control the pressure and temperature, with damping constants of 1000 for the pressure and 100 for the temperature, which implies the pressure and temperature are relaxed in a timespan of 1000 fs and 100 fs for the pressure and temperature, respectively. The pressure was damped by isotropically adjusting the simulation box. The SHAKE algorithm was used to hold the O–H bonds in the water molecules rigid as well as any other covalent bonded hydrogens and used to fix the angle of the water molecules [25].

The *NPT* simulations were conducted at 1 atm pressure and temperatures of 280 K, 300 K, and 320 K. The molecules in the simulations were heated to 500 K, then equilibrated for 1 ns. The systems were then cooled for 5 ns to the final run mid-range temperature of 300 K. These simulations were used to reach the final temperatures of 280 K and 320 K, using a 1 ns *NPT* simulation. All simulations began with a cubic box with sides of length 55 to 65 Å; following the *NPT* relaxation, they stabilized at lengths between 44 Å and 46 Å. After a 5 ns system relaxation time, the *NPT* production runs were conducted for 50 ns, collecting data every 5 ps. The *NPT* data were analyzed using the multilevel blocking method of Flyvbjerg and Petersen for the entire 50 ns production run, validating that the simulations were long enough and the data were no longer time-correlated [26].

For the 300 K systems, *NVT* simulations were also carried out starting with the “snapshot” taken from the *NPT* production run at 50 ns. After a 6 ns system relaxation time using the *NVT* ensemble, the production runs of 60 ns were carried out, with data collected every 2.5 ps. The *NVT* simulations were also analyzed using the Flyvbjerg-Petersen blocking method, incorporating the entire 60 ns of the production runs, which ensured the simulations were long enough and the data were no longer time-correlated [26]. The data analyses for the radial frequency distributions (RDFs), hydrogen bonds, water and TBP clustering were calculated using the entire 60 ns of the *NVT* runs. The RDFs and hydrogen bonding were directly calculated using VMD [13].

To further examine and more easily visualize the formation water veins for both tetrabutylphosphonium hydroxide (TBPH)–water and tetrabutylphosphonium chloride (TBPCl)–water systems, additional simulations were performed with much larger system sizes of approximately 100,000 atoms. The simulation models/methods were changed slighted as compared to the smaller simulation discussed above, for both computational efficiency and to enable future studies of cellulose systems. Most notably, these simulation use the three-site transferrable intermolecular potential (TIP3P)-pppm water model [27,28]; The TIP3P water model for these simulations of the water vein structure was used to enable future examination of systems containing cellulose bundles. The glycosylation-dependent cell adhesion molecule 2006 (GLYCAM06) force field for cellulose [29], was parameterized for the TIP3P water model. RESPA was not used [22], and instead, a single fixed timestep of 2 fs was employed. A shorter 8 Å cutoff was used for a short-range electrostatic and dispersion force for all non-bonded atoms. The PPPM method was used for the long-range electrostatics, using an accuracy of 10^−4^ [23]. Long-range dispersion forces were calculated using the Isele-Holder method [24], with a real space accuracy of 10^−3^ and a kspace accuracy of 2 × 10^−2^. These TBPH–water and TBPCl–water simulations were performed at 320 K and 360 K, respectively. The short-range electrostatic and dispersion force cutoffs were carefully reduced in the larger simulations by ensuring the system density did not change by more than 0.5% from the smaller simulations. The reduced cutoffs were able to reduce simulation time by 20% with minimal impact on structural properties. There are no notable visual differences in terms of water vein structure when comparing TIP3P simulations to the smaller TIP4P/2005 simulations at 300 K [17], however, the larger simulations provide a clearer visualization of the behavior.

Time-averaged data from the smaller simulations at 280 K, 300 K, and 320 K were used to calculate the heat capacity at constant pressure and T=300K, $c_{p}={\left(\partial H/\partial T\right)}_{p}$, via the linear approximation method [30]. The same basic approach was used to determine the thermal expansivities, $\alpha_{p}=v^{-1}{\left(\partial v/\partial T\right)}_{p}$, at T=300K.

The time-averaged mean squared displacement (TAMSD or $\bar{\delta_{i}^{2}}\left(\Delta \right)$) was utilized in addition to the mean squared displacement (MSD or $<r^{2}\left(t\right)>$), as lower water concentrations exhibited subdiffusive properties [31]. The generalized diffusion coefficient ($K_{\alpha }$) and the anomalous diffusion exponent (α) were calculated by fitting the particle-averaged TAMSDs ($<\bar{\delta^{2}}\left(\Delta \right)>$). In this system, the particle-averaged TAMSD is a smoothed and nearly identical version of the MSD, which is later explained in more detail. The ergodicity breaking parameter (χ) is also evaluated, with the definition from Grebenkov et al. [32]. The MSD, TAMSD, particle-averaged TAMSD, ergodicity breaking parameter, and anomalous diffusion equations are as follows:

(3) $$MSD=<r^{2}\left(t\right)>=\frac{1}{N}\sum_{i=1}^{N}{\left|r_{i}\left(t\right)-r_{i}\left(0\right)\right|}^{2}$$

(4) $$TAMSD=\bar{\delta_{i}^{2}}\left(\Delta \right)=\frac{1}{t-\Delta }\int_{0}^{t-\Delta }{\left|r_{i}\left(T+\Delta \right)-r_{i}\left(T\right)\right|}^{2}dT$$

(5) $$<\bar{\delta^{2}}\left(\Delta \right)>=\frac{1}{N}\sum_{i=1}^{N}\bar{\delta_{i}^{2}}\left(\Delta \right)$$

(6) $$<\bar{\delta^{2}}\left(\Delta \right)>=K_{\alpha }\Delta^{\alpha }$$

(7) $$\chi =\frac{Var[\bar{\delta_{i}^{2}}\left(\Delta \right)]}{{\left(Mean\left[\bar{\delta_{i}^{2}}\left(\Delta \right)\right]\right)}^{2}}$$

where $r_{i}$ represents the approximate center of mass of molecule *i*. The lag time, Δ, is represented in the TAMSD equations, which are the width of time windows moving along the displacement function [31]. The spacing between the time-averaged trajectories was 0.025 ns. In these calculations, the center of mass was approximated using the central P atom for the TBP^+^ cation, and the O atom for the hydroxide ion and water molecule. The center of mass approximations were sufficient, as the actual center of mass only showed minimal deviations from the utilized atoms. Specifically, for the TBP molecule, only minimal deviations averaging less than 0.5 Å occurred from the center of the P atom under energy minimized conditions for several tested configurations in Avogadro (version 1.2.0) [33], likely due to its symmetric shape and heavy central weighting. The generalized diffusion coefficient and the anomalous diffusion exponent were calculated separately for each identical ion or molecule in the simulation from 0 to 60 ns, using particle-averaged TAMSD (see Equation (6)).

While the TBPH–water mixture dissolves cellulose at room temperature, at 91.1 mol% water (40 wt% water), it is known to be thermally unstable at temperatures higher than 323K [2]. Therefore, the simulations in this work are limited to temperatures between 280 K and 320 K. At concentrations below 63 mol% water, the TBPH–water system is known to decompose, as the hydroxide reacts with the tetrabutylphosphonium (TBP^+^) cation [2,35]. The TBPH solution showed no reactivity with cellulose aside from hydrogen bonding at 93.9 mol% water (50 wt% water), which was the only concentration tested, also suggesting, that higher water concentrations would also be unreactive [2,36]. The TBPH–water solution could be reactive at low water concentration, which is discussed in more detail later; however, these non-reactive results provide viable reasons for the increased power of cellulose dissolution in the presence of water. TBPCl is sold as a pure salt, so it should be thermally stable throughout any range of water concentrations, while maintaining its cellulose solubility and co-solvent effects. In this work, the simulation range is limited from 50 to 99.97 mol% water. Data are presented at slightly below the stability range (50 to 60 mol% water), showing how the structure would look before it decays, as in the case of TBPH.

The critical water concentration range for cellulose solubility (85 to 92.5 mol% water) [2], as shown in Figure 1, has a unique set of structural and physical properties not found at other concentrations. Figure 1 also correlates these structural and chemical changes with the experimental data, which are highlighted in this experimentally optimal concentration range in the figures throughout this paper. The chemical structures of TBPH, TBPCl, and water are shown in Figure 2.

## 3. Results

### 3.1. Force Field Validation: TBPH–Water and TBPCl–Water Densities

**Figure 3:**
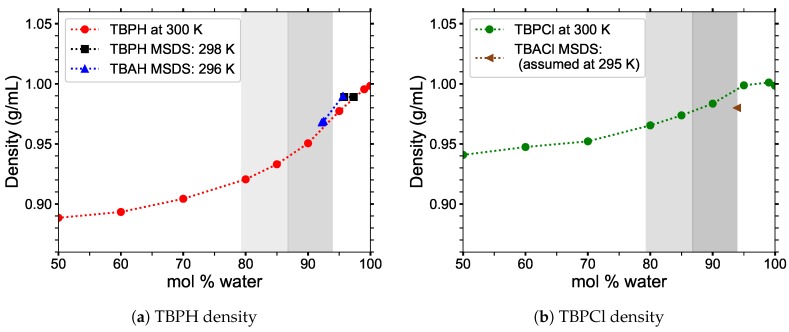
Simulated densities as a function of water concentration for *p* = 1 atm and *T* = 300 K, are plotted against material safety data sheet (MSDS) values: (**a**) TBPH density compared to MSDS values for TBPH at 60 wt% water (95.8 mol% water) [37,38], TBAH at 45 wt% water (92.2 mol% water) [39], and 60 wt% water (95.6 mol% water) [40]; (**b**) TBPCl density compared to the MSDS value for tetrabutylammonium chloride (TBACl) at 50 wt% water (93.9 mol% water) [41].

The literature values for the density of TBPH or TBPCl–water mixtures are difficult to find or non-existent. The TBPH–water IL forcefields were validated using the available material safety data sheets (MSDS), which were only available for 60 wt% water (95.8 mol% water). Tetrabutylammonium hydroxide and water (TBAH–water) IL solutions were also used to validate the force field, as it is available at lower water concentrations. TBAH–water solutions should show a similar trend as TBPH–water solutions, as they are chemically similar. The density was calculated by using the average value from the *NPT* ensemble runs. Both MSDSs confirm the trend of increasing density with water concentration and the obtained densities are reasonably close (see Figure 3). Therefore, the use of these force fields is justified.

### 3.2. Structural Properties

**Figure 4:**
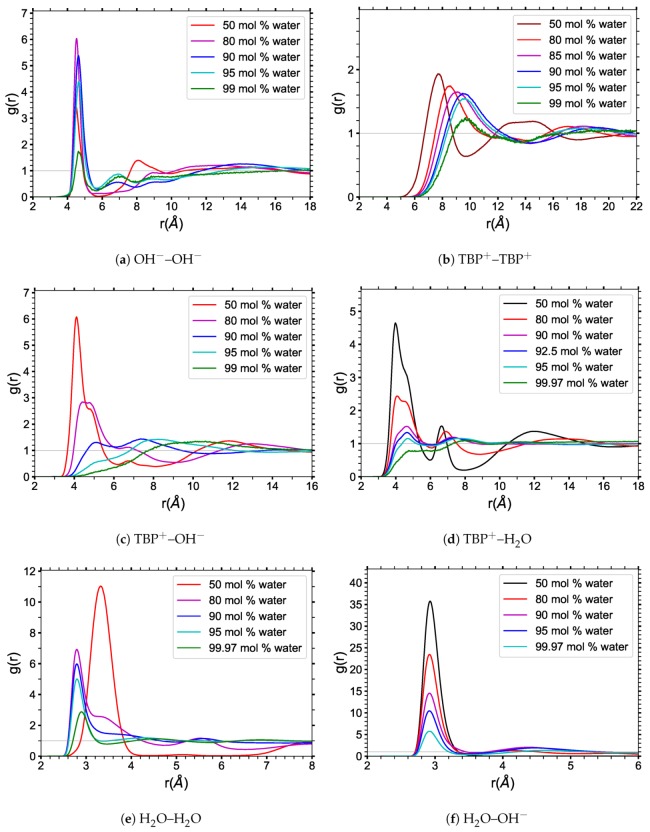
Radial distribution functions of TBPH–water at 300 K for various indicated water concentrations. The radial distribution functions are between the following molecules: (**a**) OH^−^–OH^−^; (**b**) TBP^+^–TBP^+^; (**c**) TBP^+^–OH^−^; (**d**) TBP^+^–H_2_O; (**e**) H_2_O–H_2_O; (**f**) H_2_O–OH^−^.

The radial distribution functions (RDFs) for TBPH–water were determined by using the O atom as the “center” of the hydroxide anion (OH^−^) and water, and the P atom as the “center” of the TBP^+^ cation. The RDF data were calculated and averaged for each *NVT* production run at 300 K (see Figure 4).

The TBP^+^–OH^−^ RDF (Figure 4c) shows that the hydroxide is pulled away from the cation’s P atom as the water concentration increases. This is shown by both the position and magnitude of the first major RDF peak: its location shifts from roughly 4.1 Å at 50 mol% water to about 4.6 Å at 80 mol% water, while its magnitude decreases by nearly half. Because OH^−^ can form hydrogen bond interactions with water, hydroxide ions are pulled into the void spaces created by the packing of TBP^+^; these are also the first areas that the water molecules fill. With increasing water concentration, the hydroxide continues to move away from the TBP, with a first solvation shell distance of approximately 10 Å at 99 mol% water.

The OH^−^–OH^−^ RDF (Figure 4a) for TBPH at 50 mol% water shows a distinct peak at approximately 4.5 Å that nearly doubles in size at 80 mol% water, indicating that water solvation of the hydroxide has become a dominant interaction. This also confirms that the hydroxides increasingly move to the center of the TBP cluster void space, interacting with the water molecules filling that space. The 4.5 Å peak is dominant until the water concentration reaches 95 to 99 mol% water, because of both dilution and the OH^−^–water hydrogen bonding interaction.

The TBP^+^–water RDF (Figure 4d) illustrates that from 50 mol% water, the dominant peak is at 4 Å. This wide and dominant peak indicates that some water molecules are positioned near the TBP^+^ center. These water molecules do not form a distinct structure and have some freedom to move around. At 80 mol% water concentration, the water’s nearest distance from the P atom is the same; however, the average distance increases as a result of the water cluster formation in the spaces between the TBP’s butyl arms. The second and third peaks indicate that water molecules begin to form either a structural pattern or repeatable clusters between the TBP arms. These multiple peaks arise from the ordered structure of the hydroxide–water hydrogen bonding, while the Coulombic interaction between the phosphorus and hydroxide keeps the water molecules nearby. Once the water concentration surpasses 85 mol% water, the TBP’s butyl arms no longer interlock, shifting these peaks to slightly further distances.

The TBP^+^–TBP^+^ RDF (Figure 4b) displays a transition for TBPH at 50 mol% water into a stabilized grouping of the TBP cations between 90 and 95 mol% water. This stabilized peak means that, on average, the distance between TBP^+^ cations remains fairly constant in this region. This is also the part of the region where cellulose dissolution is maximized. Dilution is responsible for the decreasing magnitude of the main peak as the water concentration increases to 99 mol% water. This is better represented in the TBP^+^ clustering data that follow.

**Figure 5:**
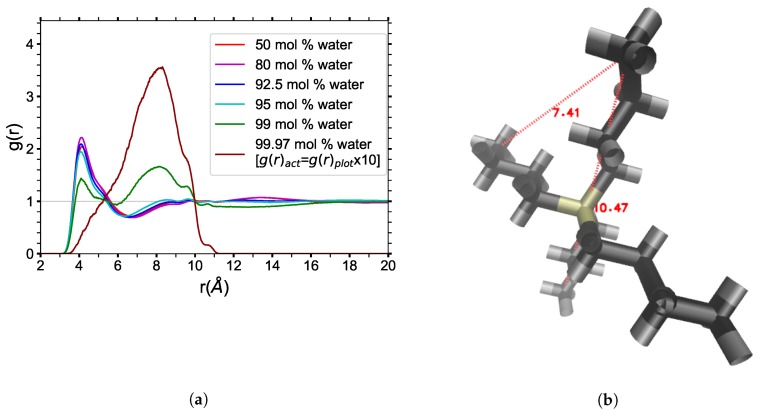
(**a**) Radial distribution functions of the end carbons on the TBP’s butyl chains (C_4_-C_4_) for the TBPH–water solution at 300 K: Note that the 99.97 mol% water data is an order of magnitude higher than the other data since it was scaled down by 1/10 to fit on the same plot; (**b**) Radial distances of the end carbons on the TBP’s butyl chains (C_4_’s), within the same molecule [13].

The RDFs of the end carbons (C_4_’s) of the TBP^+^ cation’s butyl arms have been analyzed to determine more about the conformations of the TBP^+^ cation by varying water concentration (see Figure 5). The intramolecular distribution of the arms from the central P atom is included in the RDFs, which display an increasing peak approaching 8.2 Å (see the 99.97 mol% water RDF). This peak is maximized when the TBP molecules have no other TBP neighbors.

**Figure 6:**
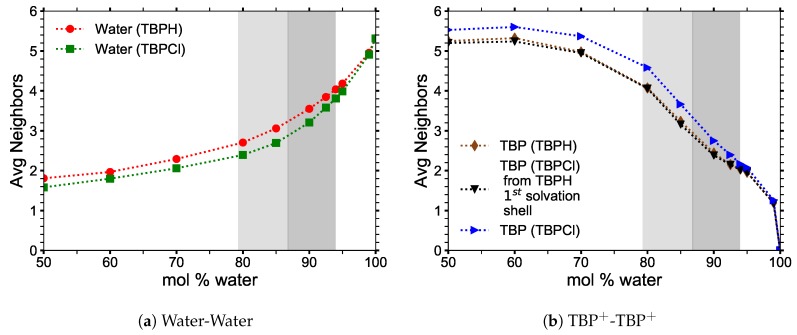
Average number of nearest molecularly identical neighbors in the largest cluster at 300 K: (**a**) the average number of waters that neighbor a water molecule; (**b**) the average number of TBPs that neighbor a TBP molecule.

**Figure A1:**
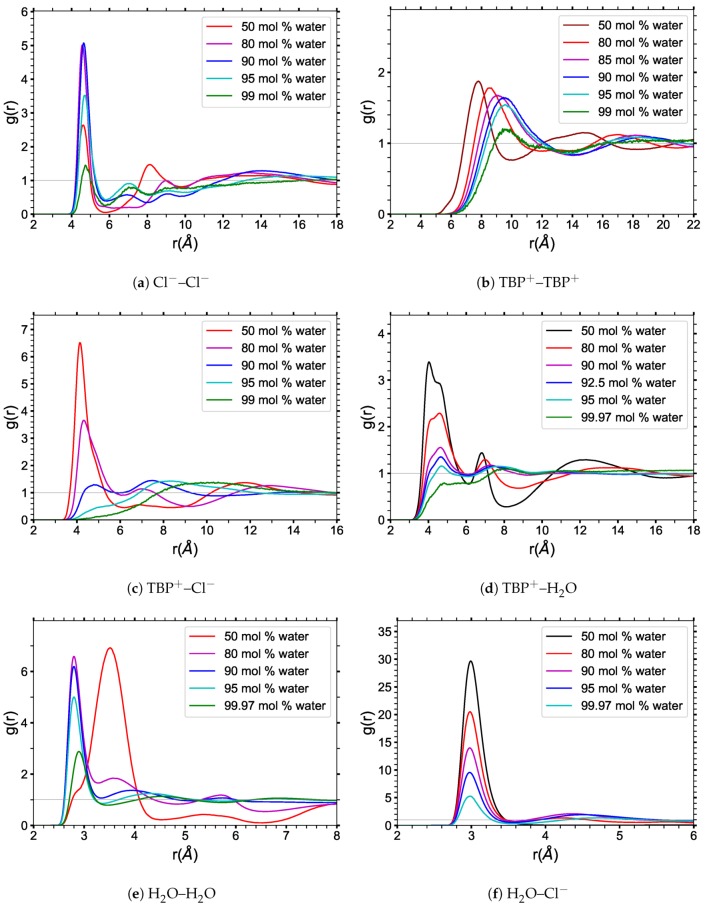
Radial distribution functions of TBPCl–water at 300 K for various indicated water concentrations. The radial distribution functions are between the following molecules: (**a**) Cl^−^–Cl^−^; (**b**) TBP^+^–TBP^+^; (**c**) TBP^+^–Cl^−^; (**d**) TBP^+^–H_2_O; (**e**) H_2_O–H_2_O; (**f**) H_2_O–Cl^−^.

**Figure A2:**
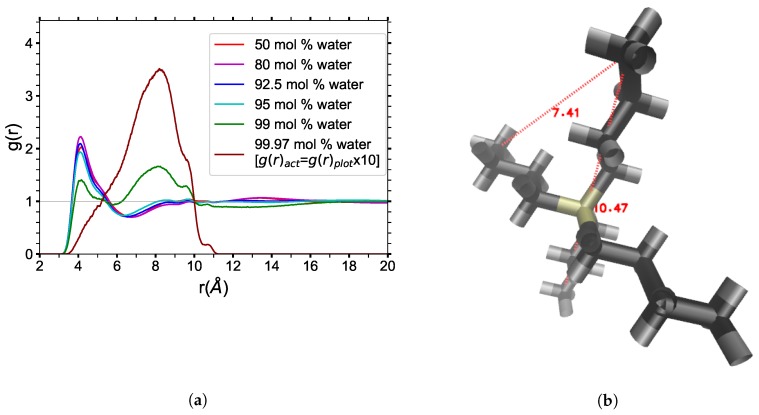
(**a**) Radial distribution functions of the end carbons on the TBP’s butyl chains (C_4_-C_4_) for the TBPCl–water solution at 300 K: Note that the 99.97 mol% water data is an order of magnitude higher than the other data since it was scaled down by 1/10 to fit on the same plot; (**b**) Radial distances of the end carbons on the TBP’s butyl chains (C4’s), within the same molecule [13].

The RDF and visualizations of pure TBPH show that the butyl arms of a TBP^+^ cation interlock with the butyl arms of other cations. As the water concentration increases, the TBP^+^’s arms spread out and the interlocking patterns morph into an end-to-end pattern (C_4_-C_4_). This pattern stabilizes between 80 and 92.5 mol% water, which is also the region where cellulose solubility is maximal (79.3 to 93.9 mol% water) [2]. As the solution is further diluted, the butyl chains begin to pull away from one another at approximately 94 mol% water. When the dilution reaches 99 mol% water, the dominant peak of the RDF is at approximately 8.2 Å, indicating that at high dilutions, there are very few neighbors, leading to spread-out conformations of the chain arms. This lack of neighboring TBP molecules at 99 mol% water is confirmed in the average number of TBP^+^ neighbors, 1.2 per cation, in the largest TBP cluster shown in Figure 6. The TBPCl–water data are strikingly similar to the TBPH–water data (see Appendix A Figure A1 and Figure A2).

### 3.3. Clustering of Water and TBP

The clustering data were collected from *NVT* simulations at 300 K. The pure TBPH and TBPCl structures were analyzed between 50 and 99.97 mol% water to determine where the pure TBP structure begins to break down, and when it no longer exists. TBP^+^ cations are considered “clustered” when the distance between the P atoms of adjacent TBP molecules is less than the radius of the first TBP^+^–TBP^+^ solvation shell in the pure TBPH or TBPCl solutions (approximately 9.1 Å for TBPH and 9.3 Å for TBPCl). The data represent the largest TBP^+^ structure in the simulation as a percentage of the total number of TBP^+^ cations in the system. At low water concentrations, almost all of the cations are part of a single cluster. Water begins to separate the TBP^+^ cations above 85 mol% water, and the cluster breaks down almost completely for concentrations over 99 mol% water.

Larger, more uniform water clusters form between 80 and 85 mol% water, and enlarge rapidly as the TBP^+^ cluster structure breaks down at 90 mol%. The water molecules form a single cluster at 90 mol% water or greater. This transformation into a single water cluster at above 90 mol% water is also observed in alkylimidazolium–water solutions at similar concentrations [10].

The average number of neighbors of TBP^+^ and water molecules in each cluster is shown in Figure 6. The number of neighbors of water remains relatively constant: each molecule has roughly two neighbors in the cluster, indicating that the water forms an essentially chainlike structure between 50 and 70 mol% water. This is a consequence of water molecules filling the void spaces in the TBP^+^ structures, which are too far apart to interact directly. The number of water neighbors increases more rapidly between 80 and 85 mol% water as the water fills all the void space and begins to break down the TBP^+^ structure, allowing the water veins to form between void spaces.

The TBP^+^ clusters decay in a much slower, steadier manner, decreasing slowly from a maximum of nearly five neighbors per cation at low water concentrations to roughly one neighbor per cation at high water concentrations. This suggests that a nearly regular tetrahedral-like arrangement for nearly pure TPB^+^ cations breaks down to what is essentially trimers as the solution becomes TBPH and TBPCl dissolved in water. This TPB^+^ trimer formation seems to level out in the maximum cellulose solubility range, especially if the first solvation shell of TBPH is used for the TBPCl trimer calculations (i.e., the first TBP^+^–TBP^+^ solvation shell in the TBPCl solution uses the 9.1 Å radii of the TBPH solution instead of the actual 9.3 Å radii of the TBPCl solution).

**Figure 7:**
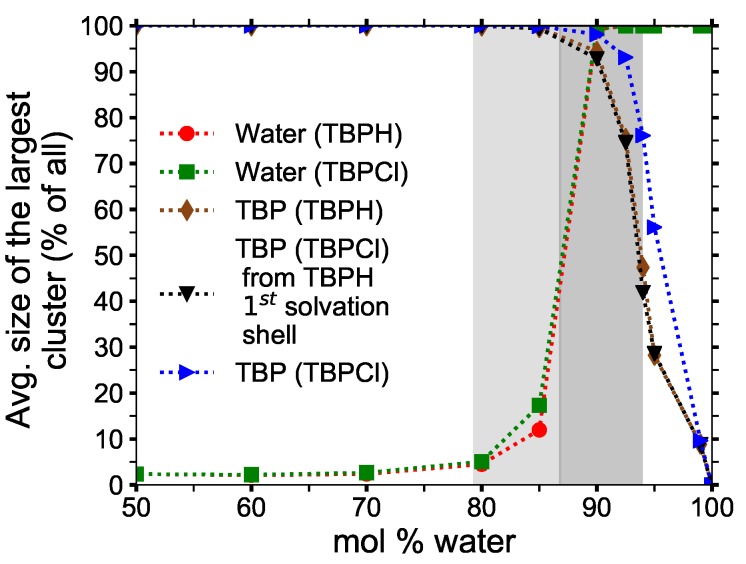
Clustering data for water and TBP^+^ ions at 300 K.

**Figure 8:**
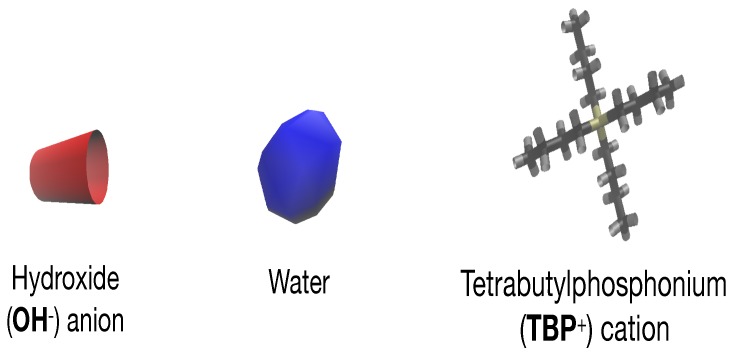
Representation of molecules in the TBPH–water solution. The blue-colored water is represented using an isosurface (called quicksurf in VMD [13]), which uses a volumetric Gaussian density map of the water to produce the observable surface. The TBP and OH molecules are represented using dynamic bonds in VMD [13]. TBP is colored tan, black and gray, for the phosphorus, carbon, and hydrogen atoms, respectively. The hydroxide is colored in red for both the oxygen and hydrogen atom.

**Figure 9:**
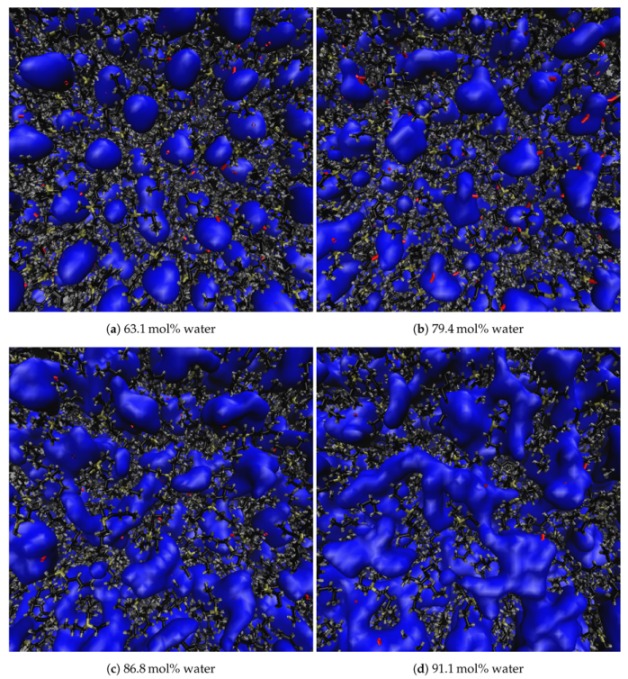
Water veins and channeling in TBPH at 320K (Part 1 of 2) [27,28]. The Figures represent the changing water structure with increasing water concentration: (**a**) 63.1 mol% water; (**b**) 79.4 mol% water; (**c**) 86.8 mol% water; (**d**) 91.1 mol% water. The blue-colored water is represented using an isosurface drawing method (called quicksurf in VMD [13]), which uses a volumetric Gaussian density map of the water to produce the observable surface. The TBP and OH molecules are represented using the dynamic bonds drawing method in VMD [13]. TBP is colored tan, black and gray, for the phosphorus, carbon, and hydrogen atoms, respectively. The hydroxide is colored in red for both the oxygen and hydrogen atom.

**Figure 10:**
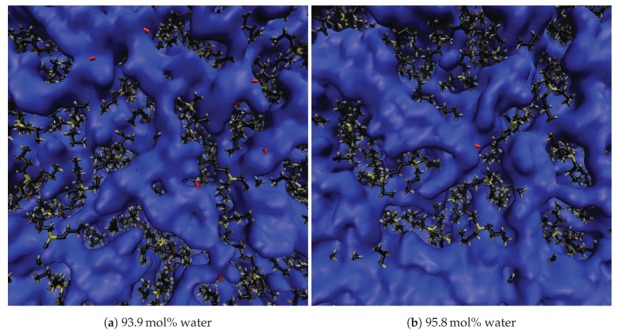
Water veins and channeling in TBPH at 320K (Part 2 of 2) [27,28]. The Figures represent the changing water structure with increasing water concentration: (**a**) 93.9 mol% water; (**b**) 95.8 mol% water. The blue-colored water is represented using an isosurface drawing method (called quicksurf in visual molecular dynamics (VMD) [13]), which uses a volumetric Gaussian density map of the water to produce the observable surface. The tetrabutylphoshonium hydroxide (TBP) and OH molecules are represented using the dynamic bonds drawing method in VMD [13]. TBP is colored tan, black and gray, for the phosphorus, carbon, and hydrogen atoms, respectively. The hydroxide is colored in red for both the oxygen and hydrogen atom.

**Figure A3:**
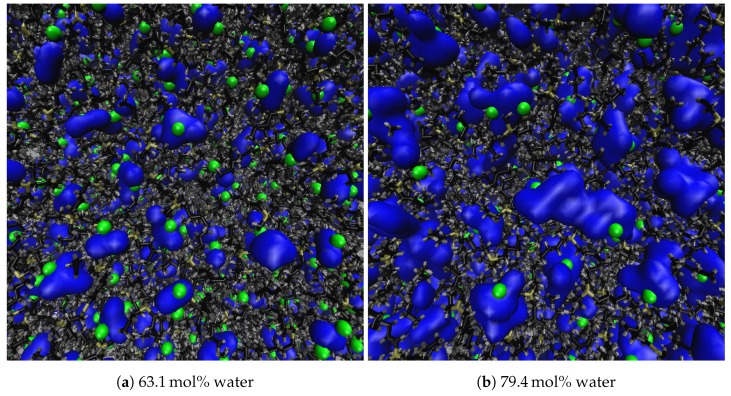

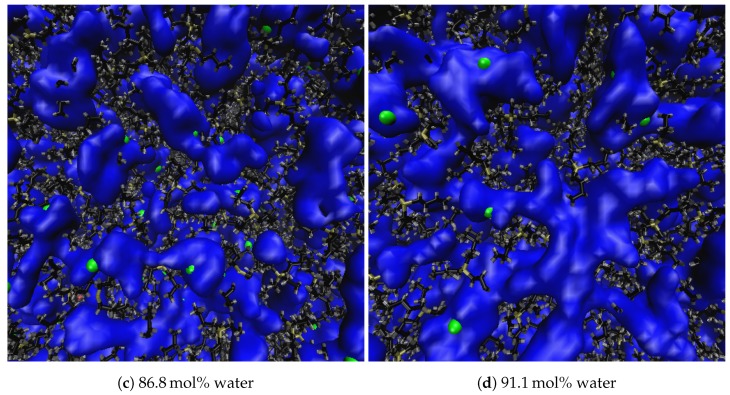
Water veins and channeling in TBPCl at 360 K (Part 1 of 2) [27,28]. The Figures represent the changing water structure with increasing water concentration: (**a**) 63.1 mol% water; (**b**) 79.4 mol% water; (**c**) 86.8 mol% water; (**d**) 91.1 mol% water. The blue-colored water is represented using an isosurface drawing method (called quicksurf in VMD [13]), which uses a volumetric Gaussian density map of the water to produce the observable surface. The TBP molecule is represented using dynamic bonds in VMD [13]. TBP is colored tan, black and gray, for the phosphorus, carbon, and hydrogen atoms, respectively. The green chloride is represented using the Van Der Walls (VDW) drawing method in VMD [13].

**Figure A4:**
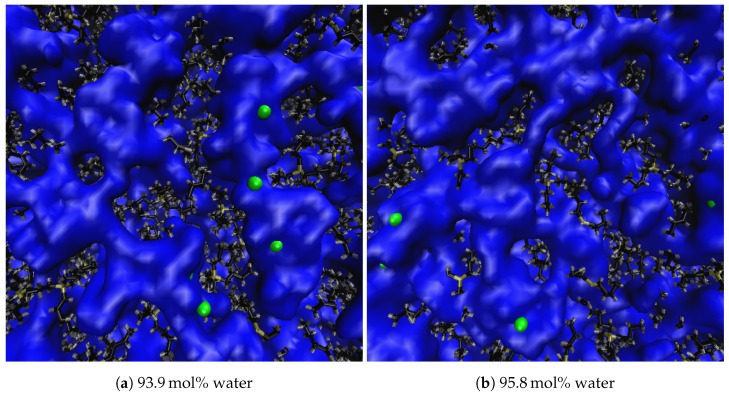
Water veins and channeling in TBPCl at 360K (Part 2 of 2) [27,28]. The Figures represent the changing water structure with increasing water concentration: (**a**) 93.9 mol% water; (**b**) 95.8 mol% water. The blue-colored water is represented using an isosurface drawing method (called quicksurf in VMD [13]), which uses a volumetric Gaussian density map of the water to produce the observable surface. The TBP molecule is represented using dynamic bonds in VMD [13]. TBP is colored tan, black and gray, for the phosphorus, carbon, and hydrogen atoms, respectively. The green chloride is represented using the Van DerWalls (VDW) drawing method in VMD [13].

**Figure A5:**
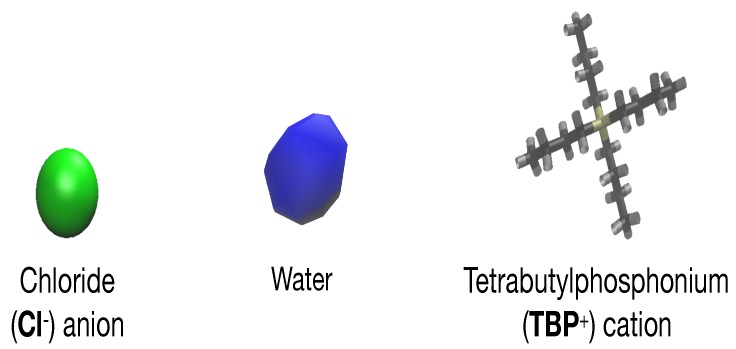
Representation of molecules for the TBPCl–water solution. The blue-colored water is represented using an isosurface drawing method (called quicksurf in VMD [13]), which uses a volumetric Gaussian density map of the water to produce the observable surface. The TBP molecule is represented using dynamic bonds in VMD [13]. TBP is colored tan, black and gray, for the phosphorus, carbon, and hydrogen atoms, respectively. The green chloride is represented using the Van Der Walls (VDW) drawing method in VMD [13].

Both the size and arrangement of the water molecules within the gaps between TBP^+^ cations evolve very slowly until 80 mol% water, beyond which the gaps grow much more rapidly. With the original TBP^+^ structure beginning to break down at 85 mol% water, veins begin to form throughout the solution (see Figure 7). The water veins remain fairly stable in shape and size at water concentrations between 85 and 92.5 mol%. Globular clusters form at 92.5 mol%, but they are not dominant. At 94 mol% the water gaps have transformed into smaller isolated globular structures, with very few, if any, water veins remaining. The structure of water changes dramatically at 95 mol%: the water clusters have completely transformed into larger globular structures with very few, if any, water veins remaining. At 99 mol% water, TBPH and TBPCl are completely dissolved in the water, which is essentially the continuous phase. As further verification of this in the TBPH–water system, snapshots of the system between 63.1 and 95.8 mol% are shown in Figure 8, Figure 9 and Figure 10, with TBP^+^ cations in tan, black and gray, the OH^−^ anions in red, and the water molecules electron shells in blue. The TBPCl–water system snapshots are shown the Appendix A (see Figure A3, Figure A4, Figure A5 ).

### 3.4. Hydrogen Bonding

**Figure 11:**
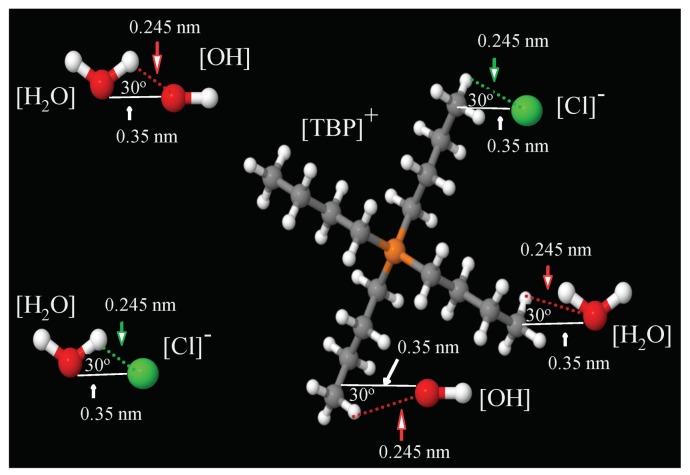
Geometric criteria for hydrogen bonds [50].

Hydrogen bonding was investigated in TBPH–water solutions between TBP^+^–water, OH^−^–water, water–water, and TBP^+^-OH^−^ pairs and TBP^+^–water, Cl^−^–water, water–water, and TBP^+^-Cl^−^ pairs in the TBPCl–water solutions. A hydrogen bond exists if the distance between the hydrogen and hydrogen acceptor is less than or equal to 2.45 Å, and the hydrogen-donor-acceptor angle is less than or equal to 30° [10,42,43,44,45,46,47,48,49]. This also requires that the donor-acceptor distance be less than or equal to 3.5 Å [10,42,43,44,45,46,47,48,49], as shown in Figure 11.

**Figure 12:**
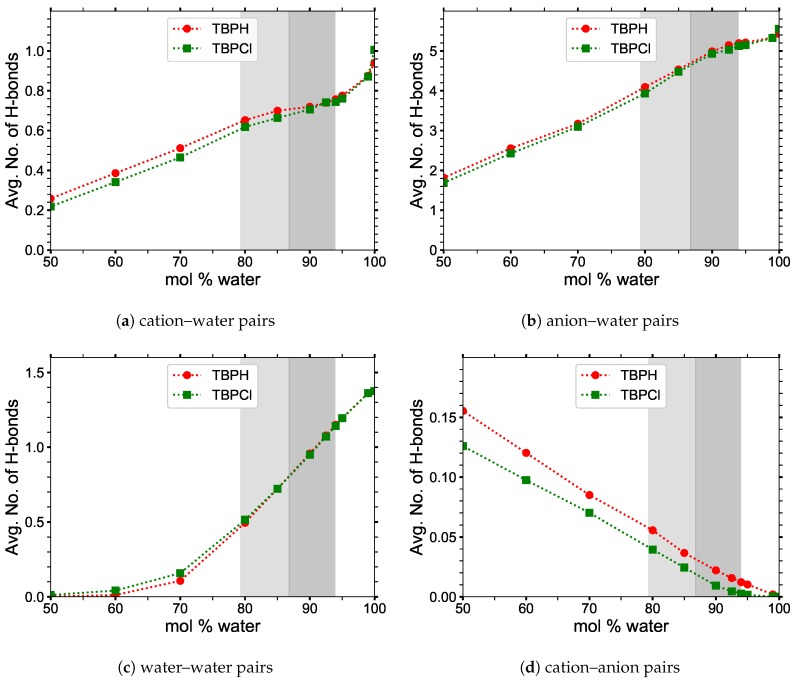
Average number of hydrogen bonds at 300 K: (**a**) cation–water pairs; (**b**) anion–water pairs; (**c**) water–water pairs; (**d**) cation–anion pairs. The first part of the labeling represents the per molecule basis (i.e., x-y represents the average number of hydrogen bonds between x and y per molecule of x).

Figure 12 shows that the hydrogen bonding of the anion–water pairs is the most dominant, with approximately 5.4 hydrogen bonds per hydroxide in 99 to 100 mol% water, in agreement with literature [51]. The least probable hydrogen bond appears to be between anion–cation pairs, decreasing in number with water concentration, because the Coulombic attraction of water is greater for the OH/Cl than for the P atom of TBP^+^. These hydroxide and chloride movements are also verified in the RDF data. As expected, the water–water hydrogen bonding follows approximately the same trend as the largest water cluster. The hydrogen bonding between TBP^+^ and water shows a defined plateau between 85 and 94 mol% water. In this concentration range, water veins are present, and the water–water hydrogen bonding, along with the anion-water interactions are more dominant. Once the veins turn into larger water clusters, at approximately 94 to 95 mol% water, the amount of TBP^+^–water hydrogen bonding begins to rise exponentially.

### 3.5. Thermodynamic Data

TPBH–water and TBPCl–water solutions with high water concentrations are liquids at room temperature. The enthalpy data for all of the concentrations were analyzed and showed smooth curves without any step changes. Thus, all the examined TBPH–water and TBPCl–water solutions were liquids, confirming that the calculations for heat capacity ($c_{p}$) and thermal expansivity ($\alpha_{p}$) are unaffected by phase changes.

#### 3.5.1. Excess Properties

**Figure 13:**
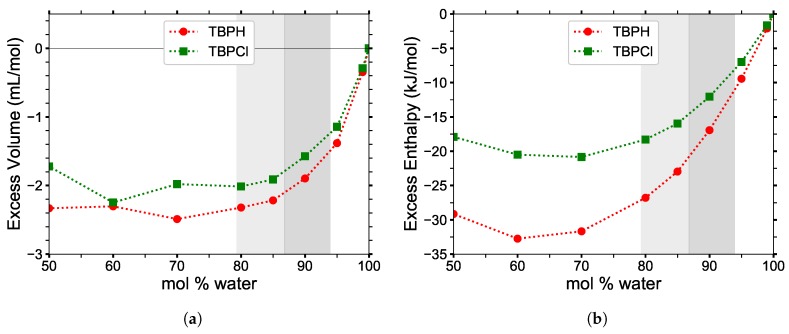
(**a**) Excess molar volume and (**b**) excess molar enthalpy of mixing of TBPH and TBPCl as a function of water concentration at *p* = 1 atm and *T* = 300 K.

Excess molar volume and excess enthalpy of mixing as a function of water concentration are shown in Figure 13. The volume corrections are relatively modest, with a maximum deviation from ideal density of only 4%. The noisiness at lower concentrations is attributed to both the initially random orientations of the water molecules as well as the rarity of water molecules at those concentrations: once trapped between the interwoven arms of the TBP^+^ structures, the water molecules remain there, yielding non-uniform water distributions throughout the solution.

The minimum in the excess enthalpy of mixing occurs when large structural changes begin to occur in the TBP^+^ arrangement. The rate of change in the excess enthalpy of mixing increases between 80 to 100 mol% water, as a result of the drastic changes in the molecular structure. The structural changes are much slower from 60 to 80 mol% water, as they originate predominantly from the anion–water hydrogen bonding. The large excess enthalpy of mixing indicates a major impact on the solution temperature, which may need to be controlled to prevent thermal instability in TBPH–water solutions. More precise experimental testing is required to determine the thermal degradation of TBPH–water solutions in the range of cellulose solubility and at varying temperatures. Using TBPCl–water solutions would eliminate this thermal instability, as the solution is known to be more stable.

#### 3.5.2. Heat Capacity and Thermal Expansivity

**Figure 14:**
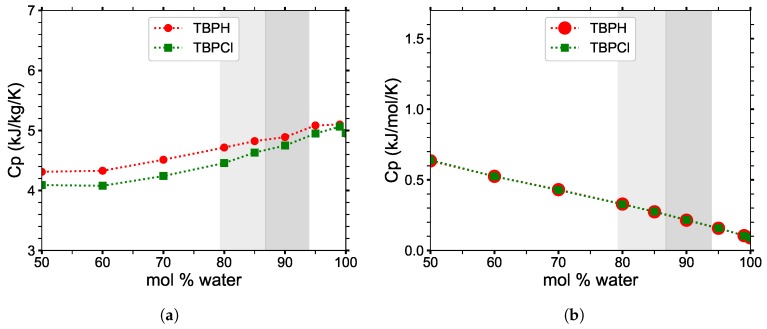
Heat capacity (*c_p_*) of TBPH–water and TBPCl–water mixtures at *p* = 1 atm and *T* = 300 K on a (**a**) per gram basis and (**b**) per mole basis, in which the values are overlapping.

On a mass basis, the heat capacity $\left(c_{p}\right)$ ranges between 4.2 and 5.1 J g^−1^ K^−1^ throughout the desired water concentration operating range (70 to 100 mol%), as shown in Figure 14 and Table 2. Given the experimental heat capacity of water, 4.184 J g^−1^ K^−1^ [52], a more precise heat capacity can be estimated for the entire concentration range. The computed ratio of experimental to simulated heat capacities for pure water is 0.82. Therefore, this can be used as a reasonable scalar correction factor to improve the simulation heat capacities. When the scalar correction factor is applied to the water concentrations, a relatively stable heat capacity of 3.4 to 4.2 J g^−1^ K^−1^ is obtained. On a molar basis, the heat capacity ($c_{p}$) is almost perfectly linear. The same scalar correction factor can be applied to the molar heat capacities to improve their accuracy.

**Figure 15:**
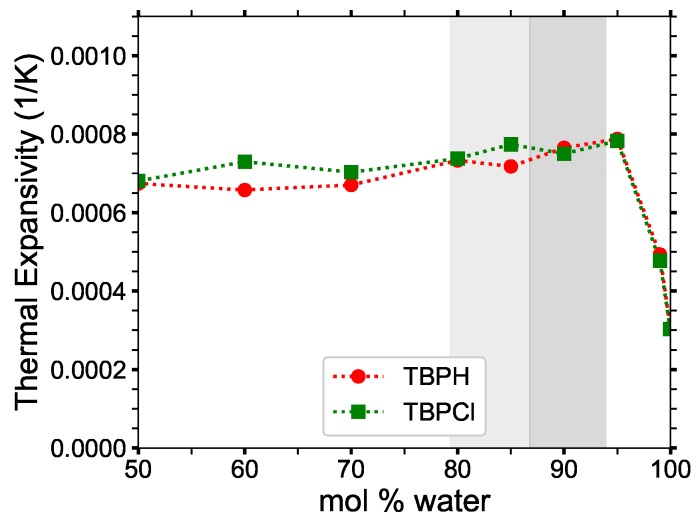
Thermal expansivity (*α_p_*) constant for TBPH–water and TBPCl–water mixtures at *p* = 1 atm and *T* = 300 K as a function of water concentration.

The thermal expansivities ($\alpha_{p}$), shown in Figure 15 and Table 2, are stable at lower concentrations, remaining at approximately 0.0007 K^−1^ up until 95 mol% water, then plummet toward the pure water thermal expansivity for higher concentrations. In the cellulose dissolving range, the thermal expansivity of TBPH–water and TBPH–water solutions are at least twice as large as that of pure water.

### 3.6. Diffusion Properties

**Figure 16:**
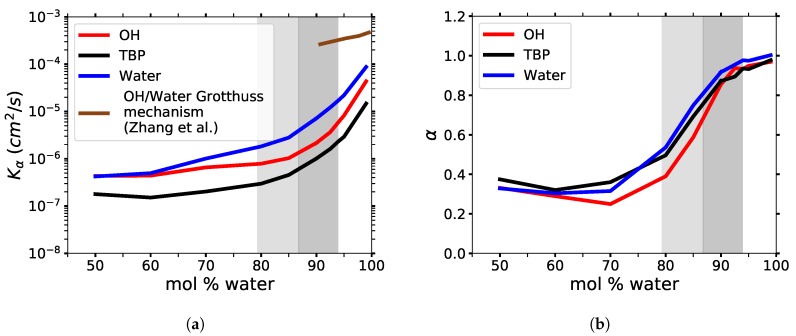
Anomalous diffusion coefficients in TBPH–water at *p* = 1 atm and *T* = 300 K as a function of water concentration: (**a**) generalized diffusion coefficient, *K_α_*; (**b**) anomalous diffusion exponent, *α*. The Grotthuss mechanism ReaxFF data was fitted to the anomalous diffusion equation with an assumed *α* value of 1 (original data from Zhang et al. [56]).

**Figure 17:**
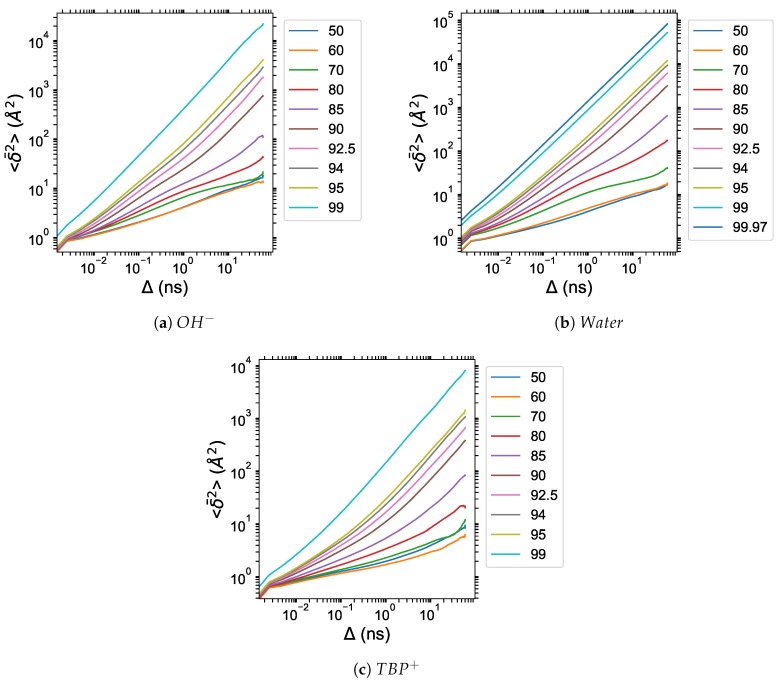
Particle-averaged time-averaged mean squared displacements (TAMSDs) of the TBPH–water solution at *p* = 1 atm and *T* = 300 K as a function of water concentration for: (**a**) *OH^−^*; (**b**) water; (**c**) *TBP*^+^.

**Figure A6:**
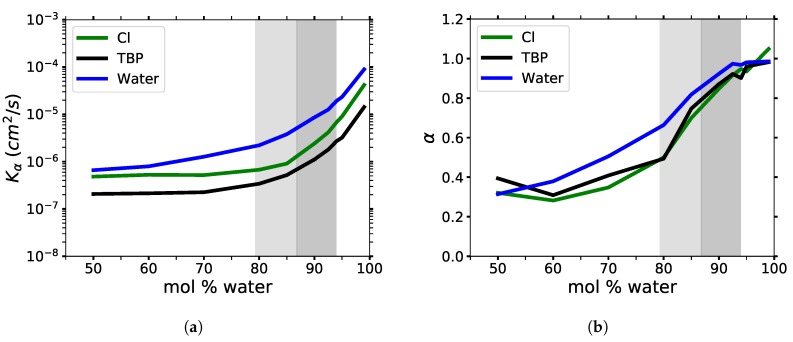
Anomalous diffusion coefficients in TBPCl–water at *p* = 1 atm and *T* = 300 K as a function of water concentration: (**a**) generalized diffusion coefficient, *K_a_*; (**b**) anomalous diffusion exponent, *α*.

The anomalous diffusion coefficients of all molecules in the TBPH–water solution are shown in Figure 16 and Table 3. The TBPCl–water solution shows similar results, and the data are available in the Appendix A (see Figure A6 and Table A1). The reported anomalous diffusion coefficients are based on the particle-averaged TAMSDs, $<\bar{\delta^{2}}\left(\Delta \right)>$, fitted to Equation (6). The α coefficient shows a large change from 80 to 92.5 mol% water, demonstrating a dramatic transition from a subdiffusive system to a near normal diffusive system (i.e., from α ≈ 0.34 to ≈ 0.93). The $K_{\alpha }$ coefficient also increases rapidly in this region and continues to increase as the system approaches pure water. In the cellulose dissolving region, the diffusion increases at least an order of magnitude. Both these coefficients indicate the average diffusion continues to increase as the system moves toward a pure co-solvent system. However, once the diffusion regime very closely approaches normal diffusion (i.e., approximately α ≥ 0.96 ), the TBPH–water solution is no longer able to dissolve cellulose. In this study, the cation, anion, and water diffusivities all trend together, in agreement with the cation and anion diffusivities from Thompson et al. [53]. The TAMSD versus time for each individual molecule displayed highly non-linear trends, resulting in anomalous diffusion at many of the water concentrations, which is not uncommon in ILs because of the added Coulombic forces (see Table 3 and Table A1, and Figure 17, Figure 19, Figure A8, Figure A9, Figure A10) [10].

**Figure 18:**
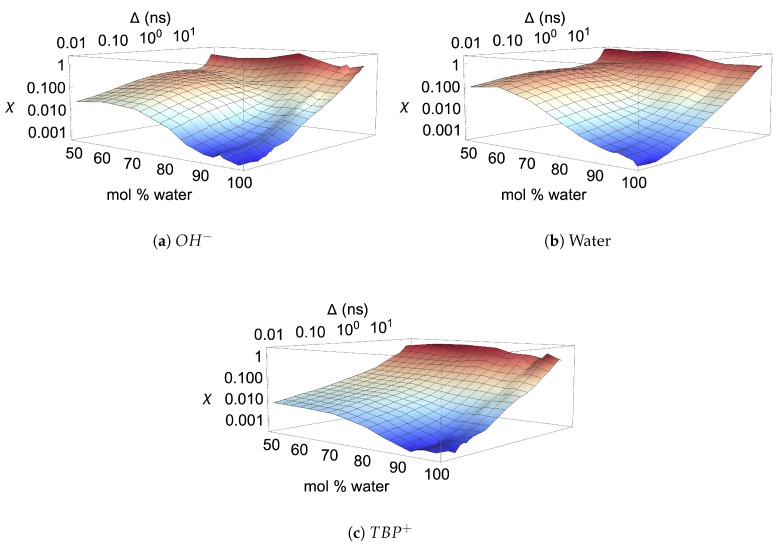
Ergodicity breaking parameter (*χ*) in TBPH–water at *p* = 1 atm and *T* = 300 K as a function of water concentration and lag time for [34]: (**a**) *OH^−^*; (**b**) water; (**a**) *TBP*^+^.

**Figure A7:**
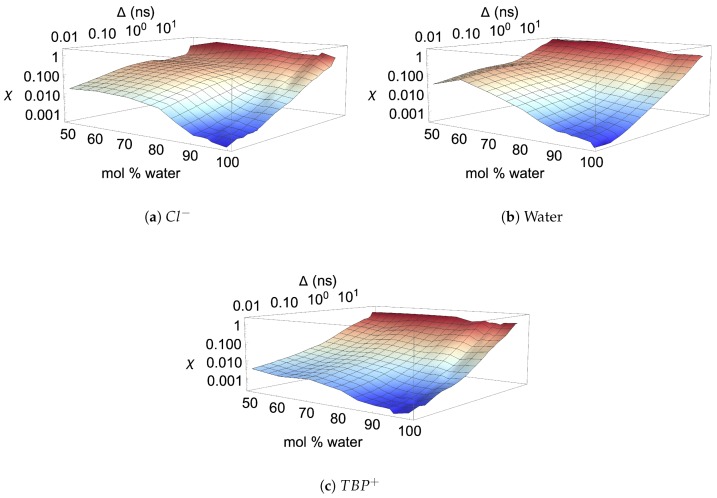
Ergodicity breaking parameter (*χ*) in TBPCl–water at *p* = 1 atm and = 300 K as a function of water concentration and lag time for [34]: (**a**) *Cl*^−^; (**b**) water; (**c**) *TBP*^+^.

The rapid change in the diffusion regime is attributed to the breaking of the TBP^+^’s interlocking arms, water vein formation, and the strong Coulombic forces. Once the TBP^+^’s interlocking arms are broken, the water, hydroxide, and chloride can “tunnel” through the water veins, further increasing their mobility [54,55]. The transition from the subdiffusive to the normal diffusive regime can be visualized in the ergodicity breaking parameter, χ, plots (see Figure 18 and Figure A7). At shorter time scales, less than a nanosecond, sufficient time-averaging in TAMSDs is available. In this timeframe, χ is at least an order of magnitude lower in the 80 to 99.97 mol% water region, also indicating that the subdiffusive region is transitioning to or is in the normal diffusive region. The anion and water have larger χ values, which plateau in the subdiffusive region, inferring they may be more affected by the trapping and caging in the subdiffusive region than the TBP molecule. At all concentrations and at longer times scales, all molecules show increasing χ values, which is in part due to a less statistical averaging from the TAMSDs in this region. This higher variance at larger time scales begins to taper off at 80 mol% water and stabilize at 92.5 mol% water, suggesting that some of the high variances could stem from faster diffusion in or along the water veins before water forms a more globular structure.

The effect of the Grotthuss mechanism on the OH^−^ and water diffusion was considered when selecting a non-reactive force field. The Grotthuss mechanism can have a large impact on the diffusion coefficients [56]. The reactive force field (ReaxFF) data from Zhang et al. are shown on the generalized diffusion coefficient plot [56], and are considered to be a likely part of the overall diffusion process, especially in the water concentration range where cellulose is soluble. While the Grotthuss mechanism likely increases the diffusivity of the TBPH–water solution, it can only do it effectively across the entire system once the TBP^+^’s interlocking arms are broken and the water pockets become connected. Therefore, the non-reactive force field that was used in this study is sufficient to demonstrate the diffusive regime changes in these solutions and thus is likely a dominant aspect in the dissolution behavior.

**Figure 19:**
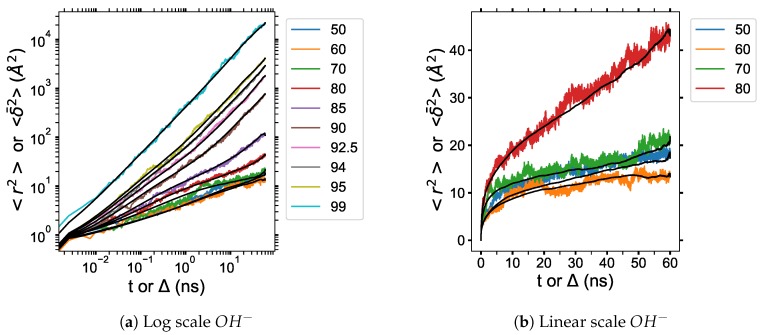
Mean squared displacement (MSD) vs. particle-averaged TAMSD of the OH ion in TBPH–water at *p* = 1 atm and *T* = 300 K as a function of water concentration: (**a**) MSD and particle-averaged TAMSD of *OH^−^* plotted on a log scale; (**b**) MSD and particle-averaged TAMSD of *OH^−^* plotted on a linear scale. The colored lines are the MSDs, and the black lines are the particle-averaged TAMSDs.

**Figure A8:**
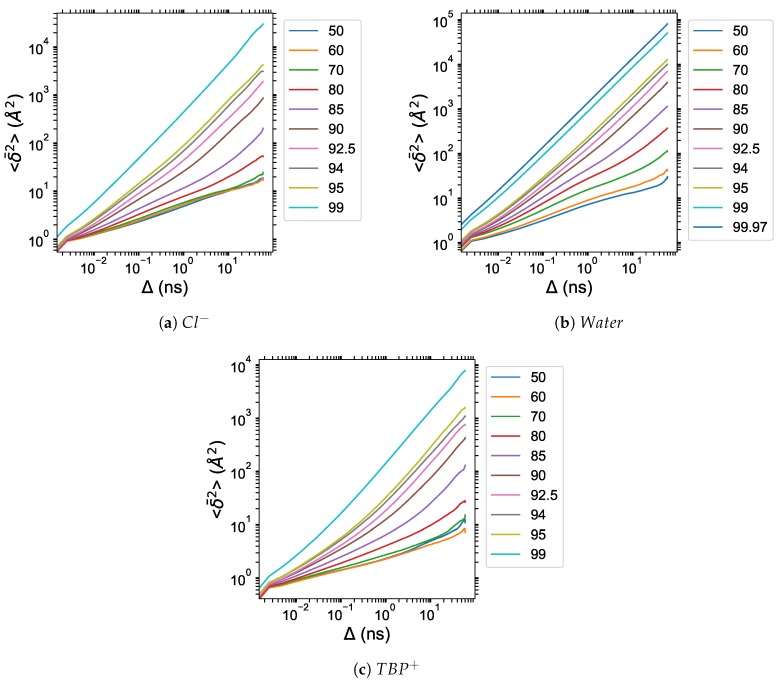
Particle-averaged TAMSDs of the TBPCl–water solution at *p* = 1 atm and *T* = 300 K as a function of water concentration for: (**a**) *Cl*^−^; (**b**) water; (**c**) *TBP*^+^.

**Figure A9:**
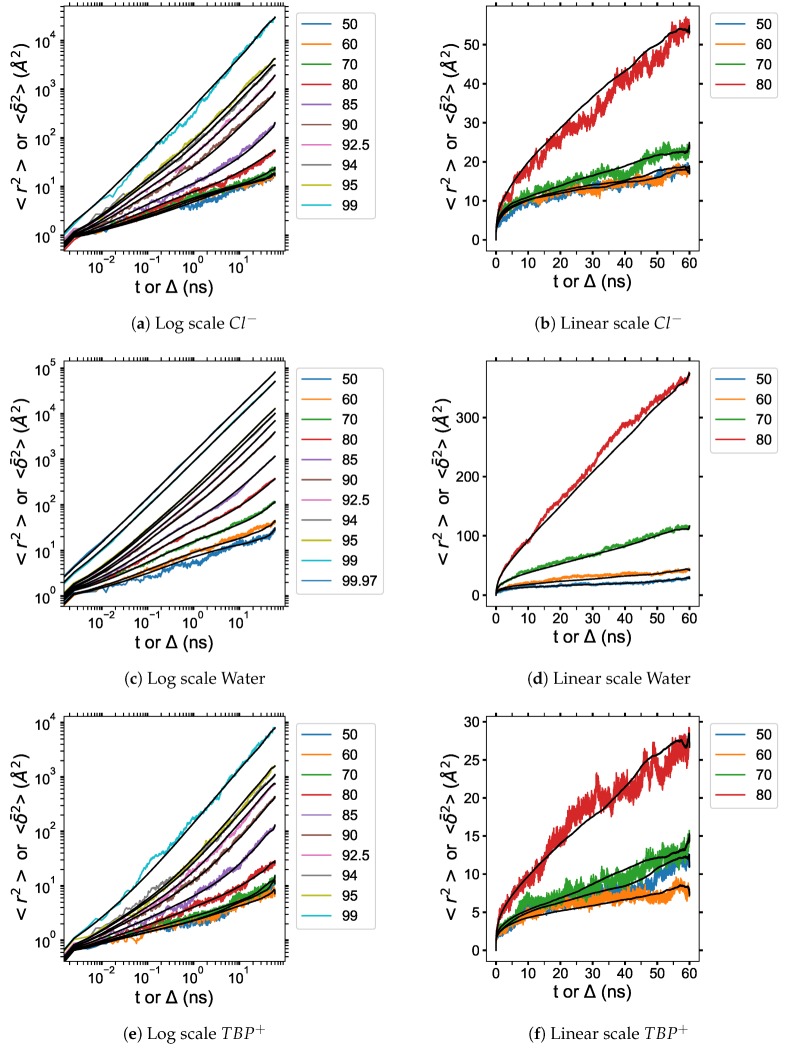
MSD vs. particle-averaged TAMSD of the water, TBP and Cl in TBPCl–water at *p* = 1 atm and *T* = 300 K as a function of water concentration: (**a**) MSD and particle-averaged TAMSD of *Cl*^−^ plotted on a log scale; (**b**) MSD and particle-averaged TAMSD of *Cl*^−^ plotted on a linear scale; (**c**) MSD and particle-averaged TAMSD of water plotted on a log scale; (**d**) MSD and particle-averaged TAMSD of water plotted on a linear scale; (**e**) MSD and particle-averaged TAMSD of *TBP*^+^ plotted on a log scale; (**f**) MSD and particle-averaged TAMSD of *TBP*^+^ plotted on a linear scale. The colored lines are the MSDs, and the black lines are the particle-averaged TAMSDs.

**Figure A10:**
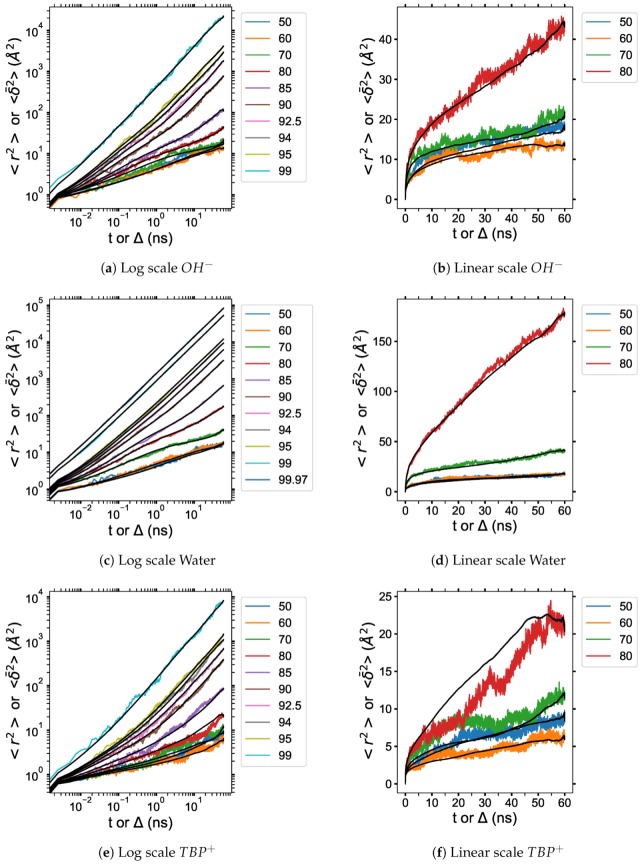
MSD vs. particle-averaged TAMSD of the water, TBP and OH in TBPH–water at *p* = 1 atm and *T* = 300 K as a function of water concentration: (**a**) MSD and particle-averaged TAMSD of *OH*^−^ plotted on a log scale; (**b**) MSD and particle-averaged TAMSD of *OH*^−^ plotted on a linear scale; (**c**) MSD and particle-averaged TAMSD of water plotted on a log scale; (**d**) MSD and particle-averaged TAMSD of water plotted on a linear scale; (**e**) MSD and particle-averaged TAMSD of *TBP*^+^ plotted on a log scale; (**f**) MSD and particle-averaged TAMSD of *TBP*^+^ plotted on a linear scale.The colored lines are the MSDs, and the black lines are the particle-averaged TAMSDs.

The particle-averaged TAMSDs for the TBPH–water system are provided in Figure 17. The particle-averaged TAMSDs for the TBPCl–water solution can be found in Figure A8 of the Appendix A. The diffusive regime can be further defined by evaluating the MSD vs. particle-averaged TAMSD, illustrated for the hydroxide in Figure 19. The other molecule’s MSD vs. particle-averaged TAMSD plot can be found in Figure A9 and Figure A10 of the Appendix A. Due to the high variance at low water concentrations from the trapping and caging by the TBP molecules, together with the MSDs and particle-averaged TAMSDs overlapping, the subdiffusion appears to stem from random and changing fractal geometries of a percolation cluster [31,32,57]. Once the solution approaches 80 to 85 mol% water, fractal geometries of a percolation cluster, or trapping and caging starts to be diminished by water vein formation, leading to the more linear particle-averaged TAMSDs found in normal diffusion. The cellulose dissolution appears to be bounded in the water vein formation region, which is essentially the percolation transition region. This bounded area is noticeable in the clustering data, the simulation ’snapshots’, and the anomalous diffusion exponent (see Figure 7, Figure 9, Figure 10, Figure 16, Figure A3, Figure A4 and Figure A6).

### 3.7. Comparison between Aqueous Solutions of Alkylimidazolium ILs and TBPH

A major difference between the cellulose dissolution capabilities of alkylimidazolium ILs and tetrabutylphosphonium hydroxide is the role that water plays at low to moderate temperatures: in alkylimidazolium ILs, water appears to act as an anti-solvent [7], while in TBPH, water acts as a co-solvent [51]. Thus, in this water concentration range the alkylimidazolium-based ILs cellulose solubility is virtually eliminated [5,10]. However, at the same time, the hydrogen bonding profile of water–water pairs is nearly identical in TBPH–water and alkylimidazolium IL–water solutions, with a sharp increase for concentrations greater than 70 mol% water. Thus, it is an interesting question of why TBPH can dissolve cellulose at higher, but not lower, water concentrations.

One of the main findings of our previous work analyzing dissolution of cellulose in ILs is that both the cation and anion played a significant role in the dissolution process [4]. First, the anion breaks some of the hydrogen bonds binding a strand to the neighboring chains in the fibril. Then, the alkylimidazolium cation acts as a “wedge,” effectively and permanently breaking the bonds and allowing for further dissolution. The simulations suggest that high water concentrations in TBPH are required to break the crippling cation structure. At lower concentrations, the TBP^+^ cations are interlocked, and thus are too hindered to wedge themselves between individual chains and the fibrils before the hydrogen bonds can be reestablished. At higher concentrations, the butyl arms begin to pull away from one another, and thus adopt a “flatter” configuration that can more easily fit between a cellulose strand and its fibril.

The diffusion trends are similar when comparing the two classes of ILs, as both show rapidly increasing diffusivities for water concentrations greater than 70 mol% water. However, this study reports the diffusion regime change from subdiffusive to near-normal diffusion in the cellulose dissolving region, which was briefly mentioned but not analyzed in the alkylimidazolium ILs diffusion studies [10,58]. One consequence of this is that the dissolution process in alkylimidazolium ILs may not depend on the diffusion regime shift. On the other hand, the diffusion regime changes where the TBP solutions can dissolve cellulose, but this could also be due to the lower temperature. Therefore, a certain level of increased diffusion with the formation of the water veins may be required for sufficient numbers of water, OH, Cl, and TBP molecules to reach the cellulose fibril to begin the dissolution process or prevent the reformation of the cellulose strands as the strands start to separate. This diffusion regime shift could also be a limiting mechanism in other IL solutions. Further verification of these hypotheses will require the direct study of cellulose in TBPCl–water systems, representing work currently underway.

## 4. Discussion

While the ability for the TBPH–water solution to dissolve cellulose with high water concentrations makes it particularly attractive as an industrial solvent, as it may not require a pre-drying step, another key economic factor for the success of any IL is its ability to support stable downstream processes. In general, TBP^+^ decreases microbial activity and thus enzymatic activity as well; however, *Chelatococcus* proteobacteria have shown good microbial activity at low [TBP][Cl] concentrations [59]. This proteobacterium, paired with TBP^+^, maybe a stable and compatible means of digesting cellulose, but more research is needed in this area [59]. TBP-based ILs are at least somewhat bio-tolerant at dilute concentrations, indicating the downstream processes could be technically and economically feasible. TBPCl–water and TBPH–water mixtures, despite the temperature limitations of the TBPH–water mixture, have significant potential as candidates in industrial processes for the extraction of cellulose from biomass. Furthermore, despite the clear correlation between the experimental observed maximum dissolution, the water vein formation, and the diffusion regime shift observed in our MD simulations, there may be other mechanistic aspects at work that cannot be captured by the use of the classical, i.e., non-reactive, AMBER forcefield [60]. Specifically, at low concentrations of water, the TBP and the hydroxide are capable of reacting. Other reactions may also occur, such as the formation a $P{\left(C_{4}H_{9}\right)}_{3}$ and $C_{4}H_{9}OH$ or any other combination of the other butyl arm carbons [35,61]. The formation of ylides and water may react in different ways with other molecules in the solution [35,61,62]. Another possible reaction could happen between the phosphorous and hydroxide forming ${\left(C_{4}H_{9}\right)}_{3}POH$ [63]. The increased length of the alkyl chains in the TBP molecule may make it less reactive than a tetramethylphosphonium or tetraethylphosphonium hydroxide, due to the steric hinderance in contacting the acidic hydrogen [62]. Additionally, the TBPCl–water solution should be more stable than the TBPH–water solution, as the chloride anion is less reactive than the hydroxide anion [3,62]. It is also possible that the mentioned reactions could contribute to the loss of cellulose dissolution in the TBPH–water solution at lower water concentrations, as the TBPCl-dimethylformamide (DMF) co-solvent mixture is capable of dissolving cellulose with the pure TBPCl IL at 343 K [3]. Additional experimental and theoretical work is required to determine all the reactions that are possible under the conditions and water concentrations in this paper. However, the water vein/channeling formation and diffusion regime changes in the TBPH/TBPCl–water systems, which correlate well with the cellulose dissolution region of the experimental TBPH–water data [2], suggest that the non-reactive, physical and property changes may be the dominant aspects with regards to cellulose dissolution at high water concentrations. This study applied classical molecular dynamics simulations, which utilize constant point charges for the atoms, instead of polarizable charges, which could mildly and more realistically alter the configurations of nearby atoms or mildly scale diffusive properties [64]. The differences in the polarizable model or the use of scaled point charges was not evaluated in this work, but would be an interesting future study for this system [64]. This study chose the existing full-scale charge force fields since they are already parameterized and computationally less expensive while providing valuable insight into the chemical system. This study was conducted using only one simulation at each concentration and temperature. Therefore, it is appropriate to mention the possibility of data sensitivity, bias, and larger errors by only using a single starting configuration or trajectory for each individual the simulations. However, each separate concentration started at a unique configuration and trajectory. Additionally, with increased computational resources, larger systems could be simulated, which could minimize the data sensitivity and error in the analysis.

## 5. Conclusions

The TBPH–water solution shows dramatic structural and diffusive changes from 80 to 100 mol% water. The pure TBP structure begins to break down at 80 to 85 mol% water, and essentially disappears when the concentration of water exceeds 95 mol%. At concentrations greater than 85 mol% water, the solution is not interwoven together by TBP^+^ cations, allowing the solution to form other structures and the cations to diffuse freely throughout the solution. The present analysis showed that small water veins begin to form everywhere in the solution between 80 and 94 mol% water, which corresponds to the cellulose solubility region (79.4 to 93.9 mol% water) [2]. Water vein formation is more prominent in the region of maximum cellulose solubility, 85 to 92.5 mol% [2]. When the concentration exceeds 94 mol% water these veins take on a more spherical or globular shape, growing in size. In this water vein region, the diffusion regime changes from a fractal subdiffusive process of a percolation cluster to a near-normal diffusive process. This effect is attributed to the decrease in TBP^+^-TBP^+^ entanglement and the dynamic coulombic force strengths. The diffusion regime shift dramatically increases the likelihood of the molecules interacting with cellulose bundles, by increasing the diffusion an by order of magnitude. The OH^−^ and Cl^−^ ions are more capable of moving into place, thus increasing the chances of breaking apart hydrogen bonds within the cellulose, and the TBP molecule is more easily able to move and act as a wedge and permanently cleave a strand from the remainder of the cellulose fibril. This allows the cellulose to dissolve in solution while inhibiting the reformation of hydrogen bonds in cellulose. We hypothesized that these combined factors of water vein formation in the water percolation region, the pure TBP structure elimination (that is, TBP^+^-TBP^+^ entanglement), and the diffusion regime shift might be the primary reasons underlying the ability of TBPH–water mixtures to dissolve cellulose at higher water concentrations, that is until the system is too diluted with water to be an effective ionic solution. The ability of TBPH to quickly dissolve high concentrations of cellulose at room temperature, within a wide range of water concentrations, makes it an ideal candidate for future full-scale industrial processes.

## Figures and Tables

**Table 1:** Compositions of TBPH/tetrabutylphosphonium chloride (TBPCl)-water systems simulated in this study.

Mole%	Total	Total	Total	Total
Water	*N*_IL_	*N*_H_2_O_	Molecules	Atoms
0.55 ^ a^	180	1	181	9,903
5.26	180	10	190	9,930
10.0	180	20	200	9,960
20.0	180	45	225	10,035
30.0	180	77	257	10,131
40.0	180	120	300	10,260
50.0	170	170	340	9,860
60.0	165	248	413	9,819
70.0	160	374	534	9,922
80.0	150	600	750	10,050
85.0	140	794	934	10,082
90.0	120	1,080	1,200	9,840
92.5^ b^	110	1,357	1,467	10,121
94.0 ^ b^	100	1,567	1,667	10,201
95.0	90	1,710	1,800	10,080
99.0	30	2,970	3,000	10,560
99.97 ^ a^	1	3,000	3,001	9,055

^a^ Infinite-dilution simulations. ^b^ Only constant volume-isothermal (*NVT*) simulations were performed at these concentrations.

**Table 2:** Heat capacities and thermal expansivities.

	TBPH–Water		
Mole%	Mass Heat Capacity	Molar Heat Capacity	Thermal Expansivity
Water	*c_p_*, J g ^−1^ K^−1^	*c_p_*, J mol^−1^ K^−1^	*α_p_*, 10^−4^ K^−1^
50.0	4.31 ± 0.17	634.7 ± 25.2	6.74 ± 0.39
60.0	4.33 ± 0.25	524.9 ± 29.8	6.57 ± 0.28
70.0	4.51 ± 0.21	430.7 ± 20.1	6.70 ± 0.26
80.0	4.72 ± 0.17	328.7 ± 12.2	7.33 ± 0.36
85.0	4.82 ± 0.16	273.7 ± 9.2	7.18 ± 0.21
90.0	4.89 ± 0.18	214.4 ± 8.1	7.65 ± 0.30
95.0	5.08 ± 0.16	157.3 ± 5.1	7.88 ± 0.23
99.0	5.10 ± 0.17	105.0 ± 3.4	4.93 ± 0.24
99.97 ^a^	4.95 ± 0.17	89.6 ± 3.0	3.07 ± 0.21
50.0	4.09 ± 0.21	639.9 ± 32.6	6.80 ± 0.22
60.0	4.08 ± 0.19	524.5 ± 24.5	7.30 ± 0.22
70.0	4.24 ± 0.19	428.2 ± 19.5	7.03 ± 0.22
80.0	4.46 ± 0.20	327.1 ± 14.5	7.38 ± 0.24
85.0	4.63 ± 0.21	275.7 ± 12.7	7.74 ± 0.36
90.0	4.75 ± 0.12	217.0 ± 5.6	7.50 ± 0.22
95.0	4.95 ± 0.20	157.7 ± 6.2	7.83 ± 0.28
99.0	5.06 ± 0.13	105.2 ± 2.7	4.77 ± 0.31
99.97 ^a^	4.96 ± 0.21	89.8 ± 3.8	3.03 ± 0.42

^a^ Infinite-dilution simulation. Data for *p* = 1 atm and *T* = 300 K. The uncertainty is calculated from the maximum slope change between the simulations at different temperatures, due to the standard deviation from the averaged parameter used in the calculations. The data represents a single simulation at each temperature.

**Table 3:** TBPH anomalous diffusion coefficients of water, TBP^+^, and OH^−^.

	*K_α_* (10^−6^ cm^2^ s^−1^)/*α* (1/*s^α^*)					
	H_2_O		TBP^+^		OH^−^	
Mole%	*K_α_*	*α*	*K_α_*	*α*	*K_α_*	*α*
50.0	0.423	0.329	0.179	0.375	0.434	0.331
60.0	0.496	0.304	0.151	0.320	0.440	0.289
70.0	1.01	0.315	0.203	0.360	0.657	0.249
80.0	1.82	0.536	0.297	0.496	0.786	0.390
85.0	2.80	0.749	0.455	0.693	1.03	0.588
90.0	7.17	0.917	1.01	0.872	2.16	0.855
92.5	12.2	0.954	1.62	0.894	3.65	0.937
94.0	17.2	0.977	2.34	0.935	5.97	0.934
95.0	22.1	0.975	2.93	0.932	8.22	0.949
99.0	87.3	1.00	14.7	0.978	43.5	0.969
99.97 ^a^	139	0.999	– –	– –	– –	– –

^a^ Infinite-dilution simulation for TBPH. Data for *p* = 1 atm and *T* = 300 K.

## Abbreviations

The following abbreviations are used in this manuscript:

AMBER	Assisted model building with energy refinement
BMIM	1-butyl-3-methylimidazolium
Cl	Chloride
DMF	Dimethylformamide
EMIM	1-ethyl-3-methylimidazolium
GLYCAM06	Glycosylation-dependent cell adhesion molecule 2006
IL	Ionic Liquid
LAMMPS	Large-scale atomic/molecular massively parallel simulator
LJ	Lennard-Jones
MD	Molecular dynamics
MSD	Mean squared displacement
MSDS	Material safety data sheet
NPT	Isobaric-isothermal
NVT	Constant volume-isothermal
O	Oxygen
OH	Hydroxide
P	Phosphorous
PPPM	Particle–particle–particle–mesh
ReaxFF	Type of reactive force field
RESPA	Reversible reference system propagator algorithms
TAMSD	Time-averaged mean squared displacement
TBA	Tetrabutylammonium
TBACl	Tetrabutylammonium chloride
TBAH	Tetrabutylammonium hydroxide
TBP	Tetrabutylphosphonium
TBPCl	Tetrabutylphosphonium chloride
TBPH	Tetrabutylphosphonium hydroxide
TIP3P	Three-site transferrable intermolecular potential
TIP4P	Four-site transferrable intermolecular potential
VMD	Visual molecular dynamics
*E*_total_	Total potential energy
*K_r_*	Potential energy bond constant
*K_θ_*	Potential energy angle constant
*K_ϕ_*	Potential energy torsion/dihedral angle constant
*n*	Number of maximums in the torsion/dihedral angle
*q_i_*	Charge of atom i
*q_j_*	Charge of atom j
*r*	Bond length
*r_ij_*	Radial distance between atoms *i* and *j*
*r*_0_	Equilibrium bond length
*γ*	Equilibrium torsion/dihedral angle
*ϵ_ij_*	Well depth of LJ potential between atoms *i* and *j*
*θ*	Bond angle
*θ*_0_	Equilibrium bond angle
*σ_ij_*	Zero of LJ potential between atoms *i* and *j*
*ϕ*	Torsion/dihedral angle
*t*	Time
Δ	Lag time
*r_i_*	Center of mass of molecule *i*
$<r^{2}\left(t\right)>$	Mean squared displacement (MSD)
$\bar{\delta_{i}^{2}}\left(\Delta \right)$	Time-averaged mean squared displacement (TAMSD) of molecule *i*
$<\bar{\delta^{2}}\left(\Delta \right)>$	Particle-averaged TAMSD
*K_α_*	Generalized diffusion coefficient
*α*	Anomalous diffusion exponent
*χ*	Ergodicity breaking parameter
*c_p_*	Heat capacity at constant pressure
*α_p_*	Thermal expansivity at constant pressure

## Appendix A

Additional visualizations of the molecular dynamics simulations include the water vein and channeling pictures for the TBPCl–water solutions, the RDFs for the TBPCl–water solutions, and additional diffusion data for the TBPH–water and TBPCl–water systems. The TBPCl water vein/channeling formation from the molecular dynamics simulations are included to provide a clearer understanding of the structural changes that occur throughout the solution, with increasing water concentrations. These TBPCl–water water vein pictures used the TIP3P-pppm water model, which differs from the rest of the TIP4P/2005 water model simulations [17,27,28]; except the other TBPH–water water vein pictures, which also used the TIP3P-pppm water model [27,28]. The diffusion data consist of the anomalous diffusion coefficients and ergodicity breaking parameters for the TBPCl–water system, and additional particle-averaged TAMSDs, and MSDs for both the TBPH–water and TBPCl–water systems.

### Appendix A.3. Diffusion Properties

**Table A1:** TBPCl anomalous diffusion coefficients of water, TBP^+^, and Cl^−^.

	*K_α_* (10^−6^ cm^2^ s^−1^)/*α* (1/*s^α^*)					
	H_2_O		TBP^+^		Cl^−^	
Mole%	*K_α_*	*α*	*K_α_*	*α*	*K_α_*	*α*
50.0	0.660	0.314	0.208	0.394	0.483	0.321
60.0	0.796	0.379	0.214	0.309	0.527	0.282
70.0	1.27	0.506	0.226	0.409	0.520	0.348
80.0	2.2	0.664	0.341	0.494	0.674	0.500
85.0	3.79	0.818	0.520	0.747	0.909	0.699
90.0	8.61	0.923	1.11	0.871	2.42	0.847
92.5	12.6	0.974	1.80	0.922	4.16	0.916
94.0	19.2	0.968	2.71	0.901	6.79	0.947
95.0	23.3	0.981	3.21	0.960	9.02	0.935
99.0	89.3	0.986	14.3	0.982	41.8	1.05
99.97 ^a^	141	0.991	– –	– –	– –	– –

^a^ Infinite-dilution simulation for TBPCl. Data for *p* = 1 atm and *T* = 300 K.
