# Erratum: Thermodynamic and Transport Properties of Tetrabutylphosphonium Hydroxide and Tetrabutylphosphonium Chloride–Water Mixtures via Molecular Dynamics Simulation

**DOI:** 10.3390/polym12040957

**Published:** 2020-04-20

**Authors:** Brad Crawford, Ahmed E. Ismail

**Affiliations:** Department of Chemical and Biomedical Engineering, West Virginia University, Morgantown, WV 26505, USA; crawfordb118@gmail.com

The authors wish to make the following changes to the published paper as listed below [1].

In the original manuscript, the chloride anion force field was missing a citation in the ‘’Simulation Methods and Details’’ section. The chloride anion force field’s citations are as follows:

The chloride ion force field was obtained from the work of Canongia Lopes et al., which developed an OPLS-AA/AMBER force field with the Lorentz–Berthelot mixing rules (note: the research by Sambasivarao et al. also derived the same constants for the OPLS-AA force field fitted to 68 unique ionic liquids) [2,3,4,5].

The labels (a) and (b) in Figure A1 were cut off. The labels are as follows: (a) is Cl^—^Cl^−^; and (b) is TBP^+^-TBP^+^”.

There are a few locations in the paper where TPB is listed. These are typos and should be TBP. Details are listed below:

- The last paragraph of the Introduction. “Here, the chemical properties of TBPH–water and”.
- The 3rd paragraph of the Section 3.3. Clustering of Water and TBP. ‘’This suggests that a nearly regular tetrahedral-like arrangement for nearly pure TBP^+^ cations breaks down to what is essentially trimers as the solution becomes TBPH and TBPCl dissolved in water. This TBP^+^ trimer formation seems to’’.
- The first paragraph of Section 3.5. Thermodynamic Data. ‘’TBPH—water and TBPCl—water solutions with high water concentrations”.

The authors apologize for any inconvenience that this has caused. These changes do not affect the scientific results. The manuscript will be updated, and the original will remain online on the article webpage https://www.mdpi.com/2073-4360/12/1/249.
